# Factors Associated With Quality of Life and Treatment Practice Among Melasma Patients in Nepal

**DOI:** 10.1155/drp/6380885

**Published:** 2026-02-09

**Authors:** Samiksha Pokhrel, Sabina Sankhi, Shishir Paudel, Anisha Chalise, Nirmal Raj Marasine

**Affiliations:** ^1^ Department of Pharmacy, CiST College, Pokhara University, Kathmandu, Nepal, pu.edu.np; ^2^ Department of Pharmacy, Shree College of Technology (SCoT), Bharatpur-12, Chitwan, Nepal; ^3^ Department of Public Health, CiST College, Pokhara University, Kathmandu, Nepal, pu.edu.np; ^4^ Center for Research on Environment, Health and Population Activities (CREHPA), Lalitpur, Nepal

**Keywords:** associated factors, melasma, Nepal, quality of life, treatment practice

## Abstract

**Background:**

Melasma is chronic, acquired hypermelanosis that commonly appears on sun‐exposed areas of the skin. Although it is asymptomatic, it can significantly affect patients’ psychosocial and emotional well‐being, ultimately reducing their quality of life. This study aimed to assess the factors associated with quality of life and treatment practices among melasma patients in Nepal.

**Methods:**

A hospital‐based cross‐sectional study was conducted among 174 melasma patients visiting Nepal Skin Hospital, Kathmandu, from March to August 2023. The Melasma Area and Severity Index (MASI) score and the Melasma Quality of Life (MELASQoL) scale were used to evaluate melasma severity and quality of life, respectively. Statistical analyses included descriptive statistics, independent *t*‐tests, one‐way ANOVA, and multiple linear regression to identify factors associated with quality of life among women with melasma.

**Results:**

The mean (±SD) MELASQoL score was 51.89. Most participants reported feeling frustrated (81.5%), embarrassed (78.8%), depressed (95.40%), and less productive (83.9%) due to melasma. Female gender, illiteracy, both current and previous OCP use, women with multiple pregnancies, lighter skin Types (III and IV), longer disease duration, and moderate MASI scores were significantly associated with higher MELASQoL values (*p* < 0.05). Sunscreen (97.7%) was the most prescribed treatment, followed by tranexamic acid (78.7%), hydroquinone (76.4%), antioxidants (71.3%), and chemical peels (69.0%).

**Conclusion:**

Melasma has a significant psychosocial impact on affected individuals in Nepal. Disease severity, use of oral contraceptive pills, number of pregnancies, disease duration, education, and skin type significantly influenced quality of life. The strong association between disease severity and MELASQoL underscores the importance of integrating psychosocial assessment into clinical management. Sunscreen followed by tranexamic acid, hydroquinone, antioxidants, chemical peels, multivitamins, and retinoids was the predominant treatment approaches.

## 1. Introduction

The skin is the largest external organ of the human body, serving as a primary protective barrier against the external environment [1]. Pigmentary disorders, including melasma, premature aging, and acne, are among the most common dermatological conditions [2]. Melasma is a chronic, acquired dermatological condition characterized by mostly symmetrical hyperpigmentation, commonly appearing on sun‐exposed areas such as the cheekbones, forehead, upper lip, chin, and nose [3].

Melasma primarily affects women, especially those of Latin American and Asian descent with Fitzpatrick skin Types IV–VI; however, men are also at risk [1, 3, 4]. The prevalence of melasma varies globally, ranging from 1% in the general population to 9%–50% in higher‐risk populations [5]. A study conducted in rural Nepal among 546 dermatological patients found melasma to be the most common pigmentary disorder and the fourth most common skin problem in the country [6].

Clinically, melasma is characterized by irregular light or dark brown macules and patches with defined margins [7]. A variety of risk factors, including genetic predisposition, UV exposure, pregnancy, oral contraceptives, hormone replacement therapy, thyroid issues, cosmetics, and certain phototoxic medications, have been linked to the condition [8].

Melanocytic hypertrophy and epidermal‐melanic hyperfunction are proposed as the primary causes of melasma. However, other studies have revealed that the interaction between keratinocytes, mast cells, genetic mutations, disrupted basement membranes, and altered vessels constitutes the main pathogenesis [1, 9, 10]. This complex pathogenesis has made it challenging to target melasma, leading to a high likelihood of recurrence and complicating its treatment.

Treatment options for melasma include tranexamic acid, oral glutathione, hydroquinone, tretinoin, azelaic acid, glycolic acid, kojic acid, 4‐n‐butylresorcinol (topical), chemical peels, microneedling, microdermabrasion, lasers, and light therapies (procedural interventions) [11, 12]. Among the available treatment options, the triple combination regimen originally described by Kligman included hydroquinone, tretinoin, and a low‐potency corticosteroid (dexamethasone). However, modern formulations frequently substitute mid‐potency corticosteroids such as mometasone or fluocinolone, which have been linked to steroid‐induced dermatoses, particularly among middle‐aged females. Although these combinations remain widely used for melasma, their potential for adverse effects and variable efficacy make them unsuitable for all skin types [12].

The World Health Organization (WHO) defines quality of life as an individual’s perception of their position in life within their cultural context, value systems, expectations, goals, morals, and concerns [1, 3]. Melasma, a facial disorder that affects the appearance of the skin, can negatively impact quality of life by lowering self‐esteem and causing feelings of embarrassment, frustration, and depression. Additionally, resistance to treatment is another factor that can lead to severe psychological distress [1, 4].

Therefore, measuring quality of life is crucial in dermatology to gain a comprehensive understanding of patients’ health status. While skin conditions may not always present direct health risks, they can significantly impact patients’ social lives and emotional well‐being [13]. Numerous studies have explored the risk factors and quality of life of patients with skin disorders worldwide; however, no study has comprehensively addressed all three aspects of melasma, particularly in Nepal. Therefore, this study aimed to assess the factors associated with quality of life and treatment practices among melasma patients in Nepal.

## 2. Methods

### 2.1. Study Design, Setting, and Population

A hospital‐based cross‐sectional study was conducted from March to August 2023 among melasma patients visiting Nepal Skin Hospital for diagnosis and management. Patients aged 18–60 years who were willing to participate were enrolled, while pregnant women, individuals with systemic causes of pigmentation or other facial dermatoses, and those taking anticonvulsants or phototoxic drugs were excluded from the study.

### 2.2. Sample Size and Sampling Technique

The required sample size was calculated using Cochran’s formula for the estimation of a proportion (*n* = *z* ^2^ *p* *q*/*d* ^2^) with a 95% confidence interval (CI) and a 5% margin of error. The prevalence estimates from a previous study (*p* = 13.03%) [13], *q* = 1–*p* = 0.87, and *z* = 1.96 were used in the calculation. Based on these parameters, the sample size was determined to be 174. Since no clear sampling frame was available, purposive sampling was used, with participants selected from those who visited the skin hospital during the data collection period.

### 2.3. Data Collection Tools and Techniques

Data collection was carried out outside the outpatient departments using a structured, self‐administered questionnaire. Participants were approached after their clinical consultation and were provided with clear instructions regarding completion of the questionnaire. For respondents who required clarification, assistance was provided to ensure accurate understanding of the questions while maintaining neutrality and avoiding response bias.

The data collection tools consisted of five comprehensive sections. The first section consisted of socio‐demographic characteristics of the participants, including age, gender, marital status, ethnicity, educational level, and occupation. The second section consisted of factors potentially influencing the development and progression of melasma. This included clinical and behavioral variables such as history of oral contraceptive pill use, number of pregnancies, skin color, eye color, duration and intensity of sun exposure, use of sunscreen during sun exposure, and age at onset of melasma.

The third section incorporated the validated Melasma Quality of Life (MELASQoL) scale [14]. The MELASQoL consists of 10 items that evaluate the impact of melasma on emotional well‐being, social life, and daily functioning. Each item is rated on a 7‐point Likert scale ranging from 1 (*not bothered at all*) to 7 (*bothered all the time*). The total score is calculated by summing individual item scores, with higher total scores indicating poorer quality of life and greater psychosocial burden. The fourth section included the Melasma Area and Severity Index (MASI) [15], a widely used clinical assessment tool that quantifies the extent and severity of melasma. The MASI score is calculated by assessing the area of involvement, darkness, and homogeneity of pigmentation across four facial regions (forehead, right malar region, left malar region, and chin). The total MASI score ranges from 0 to 48, with higher scores reflecting more severe disease. The fifth section included questions on treatment practices, such as the drug’s generic name, brand name, dosage form, strength, and frequency.

To ensure linguistic and cultural appropriateness, the questionnaire was translated from English into Nepali and subsequently back‐translated into English by independent bilingual experts. Prior to the main data collection, the questionnaire was pretested among 18 patients (representing 10% of the total sample size) at another hospital with similar patient characteristics. The internal consistency reliability of the instrument was evaluated using Cronbach’s alpha coefficient. Overall Cronbach’s alpha value was 0.88, indicating good internal consistency and reliability of the questionnaire.

### 2.4. Statistical Analysis

Data were entered into Epi‐Data Version 3.1 and analyzed using R software Version 4.0.4. The normality of continuous variables was assessed using the Shapiro–Wilk test. Descriptive statistics were used to summarize the dataset, with categorical variables presented as frequencies and percentages and continuous variables reported as mean and standard deviation.

Differences in MELASQoL scores across sociodemographic and clinical characteristics were examined using the independent *t*‐test or one‐way ANOVA, as appropriate. To identify factors associated with health‐related quality of life, multiple linear regression analyses were performed. Both unadjusted and adjusted models were constructed to determine the independent effects of relevant variables on MELASQoL scores. A *p* value < 0.05 was considered statistically significant.

### 2.5. Ethical Considerations

Ethical approval for the study was obtained from the institutional review committee of CiST (IRC‐CiST, Ref no. 75/079/080) Baneshwor, Kathmandu, Nepal, and data collection permission from Nepal Skin Hospital, Bijulibazar, Kathmandu. Patients were fully informed about the nature and purpose of the study, and written informed consent was obtained before the study began. Participation was voluntary, and participants had the right to withdraw at any stage without consequences. No financial incentives were provided, and no coercive measures were used to obtain their information. The confidentiality of patients’ data was strictly maintained throughout the study. All information was kept confidential, and anonymity was ensured. Written consent was also obtained for the publication of data.

## 3. Results

Table 1 presents the baseline characteristics of patients with melasma and their corresponding MELASQoL scores across different subgroups. The mean (±SD) MELASQoL score was 51.89. Nearly half of the participants were of age group 31–40 years (46.5%), followed by those aged 18–30 years (30.5%) and those aged over 40 years (23.0%). More than one‐third of the participants (39.1%) were engaged in service‐related occupations, followed by homemakers (20.7%) and business holders (19.0%). Regarding education, 48.3% had attained higher secondary school, 35.6% held a bachelor’s degree or above, while only 2.8% were illiterate. Most study participants (82.1%) were ever married and resided in urban areas (67.2%).

**Table 1:** Baseline characteristics of melasma patients and their MELASQoL scores (*n* = 174).

Characteristics	Total	Quality of life score		*p* value
	*n* (%)	Mean	SD	
Overall	174 (100)	51.89	9.59	
Age (years)				
18–30	53 (30.5)	51.03	10.48	0.545^a^
31–40	81 (46.5)	52.75	10.02	
> 40	40 (23.0)	51.30	7.30	
Gender				
Male	16 (9.20	42.31	13.25	< 0.01^b^
Female	158 (90.8)	52.86	8.62	
Occupation				
Business	33 (19.0)	52.87	8.30	0.02^a^
Service	68 (39.1)	50.04	12.15	
Homemaker	36 (20.7)	52.77	5.89	
Agriculture	14 (8.0)	51.57	5.87	
Unemployed	23 (13.2)	54.78	8.72	
Education				
Illiterate	5 (2.8)	59.61	4.56	0.03^a^
Primary education	10 (5.7)	51.10	6.08	
Secondary education	13 (7.5)	50.92	10.95	
Higher secondary education	84 (48.3)	49.28	6.03	
Bachelor’s and above	62 (35.6)	48.64	4.5	
Marital status				
Ever married	143 (82.1)	55.43	6.29	0.25
Never married	31 (17.9)	50.03	11.62	
Residence				
Urban	117 (67.2)	50.81	4.9	0.589
Rural	57 (32.8)	52.26	5.8	
Use of oral contraceptive pills (OCPs)				0.04^a^
Never	74 (42.5)	49.79	4.81	
Previous	66 (37.9)	53.12	6.39	
Current	34 (19.5)	54.08	8.70	
Eye color				
Medium brown	64 (36.8)	48.84	11.99	0.10^a^
Dark brown	81 (46.6)	53.44	7.89	
Green	23 (13.2)	55.04	5.87	
Chestnut	6 (3.4)	51.50	4.72	
Fitzpatrick skin type				
Type V (dark brown)	20 (11.5)	47.25	10.71	0.01^a^
Type IV (moderate brown)	109 (62.6)	52.26	9.73	
Type III (light brown/fair skin)	45 (25.9)	53.06	8.29	
Use of sunscreen during sun exposure				
Yes	100 (57.5)	51.64	10.75	0.144
No	9 (5.2)	46.11	1.20	
Sometimes	65 (37.4)	49.21	2.31	
No. of pregnancies				
≤ 1	108 (62.1)	50.85	11.14	0.04^b^
≥ 2	66 (37.9)	53.60	6.01	
Duration of melasma (in months)				
≤ 13 months	96 (55.2)	51.23	10.71	0.03^b^
≥ 14 months	78 (44.8)	53.54	8.34	
Sun exposure time (hours/day)				
≤ 2	133 (76.4)	52.03	9.71	0.351
> 2	41 (23.6)	51.37	5.56	
MASI				
Mild (≤ 8)	48 (27.6)	46.04	13.04	< 0.001^b^
Moderate (9–16)	126 (72.4)	54.12	6.73	

Abbreviation: MASI; Melasma Area and Severity Index.

^a^One‐way ANOVA.

^b^independent *t*‐test.

In terms of contraceptive use, 42.5% had never used OCPs, whereas 37.9% were previous users, and 19.5% were current users. Most participants had dark brown eyes (46.6%) or medium brown (36.8%). Fitzpatrick Skin Type IV (62.6%) was most prevalent, followed by Type III (25.9%) and Type V (11.5%). More than half (62.1%) had one or fewer pregnancies, and 44.8% had melasma for 14 months or longer. Most participants (76.4%) reported sun exposure of ≤ 2 h per day, and 57.5% used sunscreen during sun exposure. Based on MASI scoring, 72.4% had moderate melasma and 27.6% had mild disease.

MELASQoL scores across categories showed higher mean (±SD) scores among females (52.8 vs. 42.31), illiterates (59.61), and both current (54.08) and previous (53.12) OCP users. Women with two or more pregnancies (53.60 vs. 50.85) and those with longer disease duration (53.54 vs. 51.23) also reported poorer quality of life compared to their counterparts. Lighter Fitzpatrick skin Types (III and IV) showed higher scores compared to Type V. The most substantial difference was observed with melasma severity, where participants with moderate MASI had a much higher mean MELASQoL score (54.12) than those with mild melasma (46.04). Bivariate analysis also revealed a statistically significant relationship between MELASQoL score and several factors, including gender, occupation, education, use of OCPs, skin type, number of pregnancies, duration of disease, and MASI scores.

Table 2 presents the treatment practice in melasma. Sunscreen (97.7%) was the most prescribed agent among melasma patients, followed by tranexamic acid (78.7%), hydroquinone (76.4%), antioxidants (71.3%), chemical peels (69.0%), and multivitamins (52.9%) among melasma patients.

**Table 2:** Treatment practice of melasma.

Drug name	*n* (%)
Sunscreen	170 (97.7)
Tranexamic acid	137 (78.7)
Hydroquinone	133 (76.4)
Antioxidants	124 (71.3)
Chemical peel	120 (69.0)
Multivitamins	92 (52.9)
Retinoids	56 (32.2)

Table 3 presents the responses of melasma patients related to quality of life (MELASQoL). The majority reported feeling bothered by the appearance of their skin (42.0% bothered sometimes; 33.9% bothered most of the time). Similarly, 44.8% of women were frustrated about their skin most of the time, and 33.3% were frustrated sometimes. Embarrassment was also common, with 40.8% being bothered most of the time. Feeling depressed about skin condition bothered more than one‐third of participants (36.8%) most of the time. Social interaction was also affected, as 41.4% felt bothered sometimes when interacting with others, 39.1% felt their desire to be with others was affected most of the time, and 35.1% felt it was hard to show affection to others because of their skin condition. Most participants also felt unattractive (41.4%) and less productive (44.3%) due to skin discoloration most of the time. A reduced sense of freedom was reported by 47.1% of the participants most of the time.

**Table 3:** Distribution of MELASQoL responses among patients with melasma (*n* = 174).

How do you feel about	*Not bothered at all n* (%)	*Not bothered most of the time n* (%)	*Not bothered sometimes n* (%)	*Neutral n* (%)	*Bothered sometimes n* (%)	*Bothered most of the tim*e *n* %	*Bothered all the time n* (%)
The appearance of your skin condition	4 (2.3)	3 (1.7)	7 (4.0)	27 (15.5)	73 (42.0)	59 (33.9)	1 (0.6)
Frustration about your skin	3 (1.7)	3 (1.7)	5 (2.9)	21 (12.1)	58 (33.3)	78 (44.8)	6 (3.4)
Embarrassment about your skin condition	1 (0.6)	6 (3.4)	3 (1.7)	27 (15.5)	61 (35.1)	71 (40.8)	5 (2.9)
Feeling depressed about your skin condition	3 (1.7)	2 (1.1)	5 (5.7)	28 (16.1)	61 (35.1)	64 (36.8)	41 (6.3)
The effects of your skin condition on your interaction with other people	3 (1.7)	7 (4.0)	2 (1.1)	33 (19.0)	72 (41.4)	46 (26.4)	11 (6.3)
The effects of your skin condition on your desire to be with people	4 (2.3)	2 (1.1)	5 (2.9)	13 (7.5)	70 (40.2)	68 (39.1)	12 (6.9)
Your skin condition makes it hard to show affection	4 (2.3)	4 (2.3)	2 (1.1)	21 (12.1)	73 (42.0)	61 (35.1)	9 (5.2)
Skin discoloration makes you feel unattractive to others	3 (1.7)	3 (3.4)	3 (5.2)	20 (11.5)	59 (33.9)	72 (41.4)	14 (8.0)
Skin discoloration makes you feel less vital or productive	3 (1.7)	4 (2.3)	4 (2.3)	17 (9.8)	58 (33.3)	77 (44.3)	11 (6.3)
Skin discoloration affects your sense of freedom	3 (1.7)	4 (2.3)	2 (1.1)	13 (7.5)	57 (32.8)	82 (47.1)	13 (7.5)

Table 4 presents the unadjusted and adjusted linear regression analyses of factors associated with MELASQoL scores. Female participants had significantly higher MELASQoL scores compared to males (*β*: 9.81; 95% CI: 6.0–13.6). Illiterate individuals had higher quality of life impairment than those with a bachelor’s degree or above (adjusted *β*: 9.21; 95% CI: 2.0–16.4). Compared to non‐OCP users, previous users (*β*: 2.98; 95% CI: 0.2–5.8) and current users (*β*: 3.86; 95% CI: 0.9–7.0) reported poorer quality of life.

**Table 4:** Unadjusted and adjusted linear regression of factors associated with MELASQoL scores in patients with melasma.

Characteristics	Unadjusted			Adjusted		
	*β*	95% CI	*p* value	*β*	95% CI	*p* value
Gender						
Male	Ref.			Ref.		
Female	10.55	(6.8–14.3)	< 0.001^∗^	9.81	(6.0–13.6)	< 0.001^∗∗^
Occupation						
Business	Ref.			Ref.		
Service	−2.83	(−6.5–0.8)	0.12	−1.98	(−5.5–1.5)	0.27
Homemaker	−0.10	(−4.2–4.0)	0.96	0.58	(−3.4–4.6)	0.78
Agriculture	−1.30	(−6.2–3.6)	0.61	−0.72	(−5.6–4.1)	0.77
Unemployed	1.91	(−2.1–5.9)	0.35	1.15	(−2.8–5.1)	0.56
Education						
Illiterate	10.97	(3.9–18.0)	0.003^∗^	9.21	(2.0–16.4)	0.01^∗^
Primary education	2.46	(−2–6.9)	0.28	1.88	(−2.0–5.8)	0.34
Secondary education	2.28	(−1.6–6.2)	0.25	1.75	(−1.8–5.3)	0.33
Higher secondary education	0.64	(−2.2–3.5)	0.65	0.31	(−2.2–2.9)	0.81
Bachelor’s and above	Ref.			Ref.		
Oral contraceptive pills (OCPs)						
Never	Ref.			Ref.		
Previous	3.33	(0.4–6.3)	0.03^∗^	2.98	(0.2–5.8)	0.04^∗^
Current	4.29	(1.1–7.5)	0.009^∗^	3.86	(0.9–7.0)	0.01^∗^
Number of pregnancies						
≤ 1	Ref.			Ref.		
≥ 2	2.75	(0.1–5.4)	0.04^∗^	2.42	(0.1–4.9)	0.05^∗^
Duration of melasma						
≤ 13 months	Ref.			Ref.		
≥ 14 months	2.31	(0.2–4.4)	0.03^∗^	1.98	(0.1–3.9)	0.04^∗^
Skin type						
Type V	Ref.			Ref.		
Type IV	4.95	(1.1–8.8)	0.01^∗^	4.12	(0.9–7.3)	0.01^∗^
Type III	5.81	(1.6–10)	0.006^∗^	4.95	(1.3–8.6)	0.008^∗^
MASI						
Mild (≤ 8)	Ref.			Ref.		
Moderate (9–16)	8.08	(5.0–11.2)	< 0.001^∗∗^	7.45	(4.2–10.7)	< 0.001^∗∗^

*Note: β* = unstandardized regression coefficient; Ref = reference category.

Abbreviations: CI, confidence interval; MASI, Melasma Area and Severity Index.

^∗^p < 0.05 significant.

^∗∗^ *p* < 0.01 highly significant.

Women with two or more pregnancies (*β*: 2.42; 95% CI: 0.1–4.9) also demonstrated higher impairment than those with one or none. Longer duration of melasma (≥ 14 months) was also associated with higher MELASQoL scores (*β*: 1.98; 95% CI: 0.1–3.9). Individuals with skin Type III (*β*: 4.95; 95% CI: 1.3–8.6) and Type IV (*β*: 4.12; 95% CI: 0.9–7.3) showed higher scores compared to Type V. Participants with moderate MASI scores (*β*: 7.45; 95% CI: 4.2–10.7) had significantly greater quality of life impairment.

## 4. Discussion

The study assessed the factors associated with quality of life and treatment practices among melasma patients in Nepal. The findings demonstrated that melasma has a significant psychosocial burden, consistent with global evidence suggesting that pigmentary disorders markedly impair emotional, social, and functional well‐being in individuals. The mean (±SD) MELASQoL score of 51.89 obtained in the study indicates moderate to severe impairment, which aligns with the findings of other studies from Indonesia (39.97) and Brazil (34.4), thus highlighting universal psychosocial vulnerability associated with melasma, particularly among individuals with darker skin types [8, 16].

A strong association was observed between melasma severity (MASI) and quality of life, with individuals exhibiting moderate MASI scores reporting significantly higher MELASQoL scores, indicating substantially poorer quality of life. This finding aligns with evidence from a systematic review and meta‐analysis [3] and Indonesian study [8], both of which concluded that increasing disease severity is a key predictor of deteriorating psychosocial well‐being. Additionally, the chronic and recurrent nature of melasma often makes treatment challenging and may further exacerbate emotional distress among affected individuals.

In the study, sociodemographic characteristics, including gender and education, illustrated significant association with MELASQoL scores. Compared to males, females experienced greater quality of life impairment with melasma, which is in line with studies from South Africa [1] and Pakistan [17], reflecting the heightened social and cultural emphasis on women’s facial appearance in many societies. Likewise, the higher score and highly impaired quality of life in illiterate individuals may be associated with lower awareness of treatment options and deleted healthcare‐seeking behaviors in them, a pattern similarly identified in a Brazilian study [18].

Moreover, reproductive and hormonal factors were also found to be linked to poorer quality of life among patients, as both the previous and current OCP users were reported with higher MELASQoL scores. This is supported by the role estrogen and progesterone receptors play in melasma pathogenesis [19]. In the study, women with two or more pregnancies exhibited poorer quality of life, supporting findings from a Turkish study, which reported pregnancy‐ and contraceptive‐use‐related hyperpigmentation as a major contributor of psychosocial burden and emotional stress in women [7].

Skin type was another significant factor, with individuals having Fitzpatrick Types III and IV reporting poorer quality of life, findings parallel to those from the Brazilian studies [5, 19]. Longer disease duration was also associated with poorer quality of life, consistent with previous findings that chronic melasma contributes to long‐term emotional stress, loss of confidence, and persistent dissatisfaction with appearance [13]. The chronic nature and resistance to treatment heightens frustration, which is evident in the study wherein over 80% of patients reported feeling frustrated, embarrassed, and depressed due to their skin condition.

Regarding treatment practices, sunscreen was the most frequently prescribed intervention, consistent with international recommendations that emphasize photoprotection as the cornerstone of melasma management. Evidence suggests that the combined use of broad‐spectrum sunscreen with topical or systemic agents can significantly improve both MASI and MELASQoL scores [20], supporting the treatment approach observed in this cohort. The widespread use of hydroquinone, tranexamic acid, and antioxidants further reflects global clinical trends favoring combination therapy for optimal outcomes [11, 15]. Furthermore, the notable use of chemical peels highlights the growing adaptation of procedural treatment for melasma management in Nepal.

This study is among the few in Nepal to comprehensively evaluate melasma severity, quality of life, and treatment practices using validated tools such as MELASQoL and MASI, enhancing the reliability of the findings. However, several limitations should be acknowledged. The cross‐sectional design limits causal inference, and the use of purposive sampling from a single hospital may introduce selection bias, restricting generalizability to the broader melasma population. Self‐reported data may be subject to recall or social desirability bias. Additionally, the absence of follow‐up data prevents assessment of long‐term treatment effectiveness and changes in psychosocial well‐being over time. The study did not account for seasonal variation, prior treatment duration, or recurrence patterns, all of which may influence patients’ perceived quality of life.

## 5. Conclusion

The study demonstrates that melasma imposes a significant psychosocial burden on affected individuals in Nepal, with quality of life significantly influenced by disease severity, hormonal and reproductive factors, education level, disease duration, and skin type. Individuals with moderate MASI scores, longer disease duration, those using OCPs, women with multiple pregnancies, and skin types III and IV experienced poorer quality of life. Sunscreen was the widely prescribed treatment, followed by tranexamic acid, hydroquinone, antioxidants, chemical peels, and retinoids, reflecting current global clinical practice. The strong association between MASI and MELASQoL underscores the need for clinicians to address both clinical and psychosocial aspects when managing melasma. Longitudinal studies are recommended to better understand treatment effectiveness and long‐term psychosocial impact.

## Author Contributions

Samiksha Pokhrel: conceptualization, methodology, data curation, investigation, validation, and project administration; Sabina Sankhi: methodology, formal analysis, visualization, writing–original draft, and writing–review and editing; Shishir Paudel and Anisha Chalise: validation, methodology, and writing–review and editing; Nirmal Raj Marasine: conceptualization, methodology, validation, formal analysis, supervision, visualization, resources, writing–original draft, and writing–review and editing.

## Funding

No funding was received for this manuscript.

## Conflicts of Interest

The authors declare no conflicts of interest.

## Data Availability

The raw data used to support the findings of this study are made available from the corresponding author upon reasonable request.

## References

[1] Mpofana N. , Paulse M. , Gqaleni N. et al., The Effect of Melasma on the Quality of Life in People With Darker Skin Types Living in Durban, South Africa, International Journal of Environmental Research and Public Health. (2023) 20, no. 22, 10.3390/ijerph20227068. PMC10671852 37998299

[2] Amatya B. , Jha A. K. , and Shrestha S. , Frequency of Different Types of Facial Melanoses Referring to the Department of Dermatology and Venereology, Nepal Medical College and Teaching Hospital in 2019, and Assessment of Their Effect on Health-Related Quality of Life, BMC Dermatology. (2020) 20, 1–7, 10.1186/s12895-020-00100-3. 32746823 PMC7398190

[3] Zhu Y. , Zeng X. , Ying J. , Cai Y. , Qiu Y. , and Xiang W. , Evaluating the Quality of Life Among Melasma Patients Using the MELASQoL Scale: A Systematic Review and Meta-Analysis, PLoS One. (2022) 17, no. 1, 10.1371/journal.pone.0262833. PMC8794204 35085327

[4] Rahman A. , Basit A. , Mohsin S. , Ahmed N. , Tahir M. , and Ishfaq A. , Quality of Life of Melasma Patients in Pakistan, Pakistan Armed Forces Medical Journal. (2022) 72, no. 1, 307–310, 10.51253/pafmj.v72i1.7401.

[5] Majid I. and Aleem S. , Melasma: Update on Epidemiology, Clinical Presentation, Assessment, and Scoring, Journal of Skin and Stem Cell. (2021) 8, no. 4, 10.5812/jssc.120283.

[6] Walker S. , Shah M. , Hubbard V. , Pradhan H. , and Ghimire M. , Skin Disease Is Common in Rural Nepal: Results of a Point Prevalence Study, British Journal of Dermatology. (2008) 158, no. 0, 334–338, 10.1111/j.1365-2133.2007.08107.x, 2-s2.0-38349189458. 17711533

[7] Türkmen H. and Yörük S. , Risk Factors of Striae Gravidarum and Chloasma Melasma and Their Effects on Quality of Life, Journal of Cosmetic Dermatology. (2023) 22, no. 2, 603–612, 10.1111/jocd.14783. 35037372

[8] Jusuf N. K. , Putra I. B. , and Mahdalena M. , Is There a Correlation Between Severity of Melasma and Quality of Life?, Open Access Macedonian Journal of Medical Sciences. (2019) 7, no. 16, 2615–2618, 10.3889/oamjms.2019.407. 31777617 PMC6876811

[9] Maranzatto C. F. P. , Miot H. A. , Miot L. D. B. , and Meneguin S. , Psychometrican Analysis and Dimensional Structure of the Brazilian Version of Melasma Quality of Life Scale (MELASQoL-BP), Anais Brasileiros de Dermatologia. (2016) 91, no. 4, 422–428, 10.1590/abd1806-4841.20165014, 2-s2.0-84984799903. 27579735 PMC4999098

[10] Torres-Álvarez B. , Mesa-Garza I. G. , Castanedo-Cázares J. P. M. et al., Histochemical and Immunohistochemical Study in Melasma: Evidence of Damage in the Basal Membrane, American Journal of Dermatopathology. (2011) 33, 291–295, 10.1097/DAD.0b013e3181ef2d45, 2-s2.0-79955522375. 21317614

[11] Mpofana N. , Chibi B. , Visser T. et al., Treatment of Melasma on Darker Skin Types: A Scoping Review, Cosmetics. (2023) 10, no. 1, 10.3390/cosmetics10010025. PMC10416367 37563624

[12] Basit A. , Rahman A. , and Uddin R. , Oral Tranexemic Acid With Triple Combination Cream (Flucinolone + Hydroquinone + Tretinoin) Versus Triple Combination Cream Alone in Treatment of Melasma, Journal of Ayub Medical College Abbottabad-Pakistan. (January 2021) 33, no. 2, 293–298, https://jamc.ayubmed.edu.pk/index.php/jamc/article/view/8559/3107. 34137548

[13] Pudasaini P. and Neupane S. , An Observational Study to Evaluate Quality of Life in Patients With Melasma in a Tertiary Level Hospital of Pokhara, Nepal Journal of Dermatology, Venereology and Leprology. (2021) 19, no. 1, 37–41, 10.3126/njdvl.v19i1.35047.

[14] Balkrishnan R. , McMichael A. J. , Camacho F. T. et al., Development and Validation of a Health-Related Quality of Life Instrument for Women With Melasma, British Journal of Dermatology. (2003) 149, no. 3, 572–577, 10.1046/j.1365-2133.2003.05419.x, 2-s2.0-0142120540. 14510991

[15] Kimbrough-Green C. K. , Griffiths C. E. M. , Finkel L. J. et al., Topical Retinoic Acid (Tretinoin) for Melasma in Black Patients. A Vehicle-Controlled Clinical Trial, Archives of Dermatology. (1994) 130, no. 6, 727–733, 10.1001/archderm.1994.01690060057005, 2-s2.0-0028334306. 8002642

[16] Ikino J. K. , Nunes D. H. , Silva V. P. , Fröde T. S. , and Sens M. M. , Melasma and Assessment of the Quality of Life in Brazilian Women, Anais Brasileiros de Dermatologia. (2015) 90, no. 2, 196–200, 10.1590/abd1806-4841.20152771, 2-s2.0-84926367429. 25830989 PMC4371668

[17] Akhunzada W. A. , Luqman N. , Luqman A. , Khalid M. , and Jam S. , Melasma: Quality of Life in Patients, Professional Medical Journal. (November 2017) 24, no. 12, 1899–1903, 10.17957/TPMJ/17.4206.

[18] Pollo C. F. , Miot L. D. , Meneguin S. , and Miot H. A. , Factors Associated With Quality of Life in Facial Melasma: A Cross‐Sectional Study, International Journal of Cosmetic Science. (2018) 40, no. 3, 313–316, 10.1111/ics.12464, 2-s2.0-85050010589. 29734511

[19] Tamega A. D. , Miot H. A. , Moco N. P. , Silva M. G. , Marques M. E. , and Miot L. D. , Gene and Protein Expression of Oestrogen‐β and Progesterone Receptors in Facial Melasma and Adjacent Healthy Skin in Women, International Journal of Cosmetic Science. (2015) 37, no. 2, 222–228, 10.1111/ics.12186, 2-s2.0-84924080027. 25439299

[20] Sarkar R. , Ghunawat S. , Narang I. , Verma S. , Garg V. K. , and Dua R. , Role of Broad‐Spectrum Sunscreen Alone in the Improvement of Melasma Area Severity Index (MASI) and Melasma Quality of Life Index in Melasma, Journal of Cosmetic Dermatology. (2019) 18, no. 4, 1066–1073, 10.1111/jocd.12911, 2-s2.0-85065212111. 31033184

